# Editorial: Enteric Viruses in Food: Survival and Inactivation Methods

**DOI:** 10.3389/fmicb.2021.722368

**Published:** 2021-07-22

**Authors:** Walter Randazzo, Sophie Zuber, Doris H. D'Souza, Gloria Sánchez

**Affiliations:** ^1^Department of Preservation and Food Safety Technologies, Institute of Agrochemistry and Food Technology (IATA-CSIC), Valencia, Spain; ^2^Nestlé Research, Institute of Food Safety and Analytical Science, Lausanne, Switzerland; ^3^Department of Food Science, University of Tennessee-Knoxville, Knoxville, TN, United States

**Keywords:** enteric viruses, food safety, inactivation, food processing, molecular methods

Foodborne outbreaks caused by human enteric viruses, particularly human norovirus and hepatitis A virus (HAV), are of concern to global public health and agriculture. Despite being a major cause of foodborne outbreaks in high-income countries, human enteric viruses have received comparatively less attention than other foodborne pathogens (WHO, 2015). Since enteric viral pathogens are mainly transmitted by the fecal-oral route, any food along the food chain is susceptible to viral contamination, with shellfish, leafy greens, and berries being the products most frequently associated with viral foodborne outbreaks (EFSA and ECDC, 2021). The low infectious dose of most human enteric viruses, together with their high stability in the environment, make them extremely infectious and highly transmissible (Teunis et al., 2008; Atmar et al., 2014). From a food safety perspective, the prevention of viral contamination by good agricultural, hygienic, and manufacturing practices remains the main pursued strategy (Barclay et al., 2014). Food processing technologies help to control viral contamination of raw food products and to minimize the risk of cross-contamination and/or recontamination, in line with the principles of the hurdle technology. However, human enteric viruses tend to be more resistant to inactivation by traditional and emerging food manufacturing processes than foodborne bacteria.

This Research Topic comprises five original research articles focusing on different treatments to inactivate the main foodborne viruses, i.e., human norovirus, HAV and hepatitis E virus (HEV). Nasheri et al. explored high-pressure processing (HPP) as a promising non-thermal technology for HEV inactivation in pork pâté. Based on an experimental design using cell culture, HPP treatments at 600 MPa partially inactivated HEV. Fuentes et al. investigated the inactivation of HAV and norovirus in clams treated by heat using cell-culture and viability PCR. Interestingly, results showed that data from viability PCR matched infectivity data obtained by cell-culture methods, finally providing an additional rapid molecular tool for risk assessment. Also dealing with shellfish contamination, Sharp et al. demonstrated that depuration failed to remove noroviruses in mussels and that *Escherichia coli* is not a good indicator for assessing their virological quality.

Moving to fresh vegetables as another risky food commodity, Ha et al. evaluated slightly acidic electrolyzed water (SAEW) for disinfecting cabbage contaminated with norovirus. By using data modeling, the authors demonstrated that physicochemical parameters of SAEW play a key role on virus inactivation kinetics.

Filipić et al. investigated the viral inactivation potential of cold atmospheric plasma using Pepper Mild Mottle Virus (PMMoV) as viral surrogate. The findings of this study demonstrated cold atmospheric plasma as suitable treatment for viral inactivation in water samples.

Viral stability on food contact and food surfaces was evaluated by Trudel-Ferland et al. using inactivated HAV on stainless steel, polyvinyl chloride and blueberries. Testing a wide temperature range, the authors observed a considerable persistence of HAV RNA over time on non-porous surfaces and foods.

In summary, these articles present original research on several strategies effective in controlling human enteric viruses along the food chain. They provide valuable data on the performance of different technologies able to reduce viral contamination in water and food. Furthermore, as standard molecular methods such as ISO 15216 are currently applied for viral detection, the studies included within this Research Topic emphasize the need to develop alternative approaches for distinguishing non-infectious and infectious viruses. In summary, these contributions represent a step forward in the assessment of the microbiological risk posed by enteric viruses and in the protection of consumers against the exposure to these viruses along the food chain.

Lastly, the editors of this topic would like to thank all authors and reviewers for their contributions to the present collection.

## Author Contributions

All authors listed have made a substantial, direct and intellectual contribution to the work, and approved it for publication.

## Conflict of Interest

The authors declare that the research was conducted in the absence of any commercial or financial relationships that could be construed as a potential conflict of interest.

## References

[B1] Atmar R. L. Opekun A. R. Gilger M. A. Estes M. K. Crawford S. E. Neill F. H.. (2014). Determination of the 50% human infectious dose for norwalk virus. J. Infect. Dis. 209, 1016–1022. 10.1093/infdis/jit620 24253285 PMC3952671

[B2] Barclay L. Park G. W. Vega E. Hall A. Parashar U. Vinjé J.. (2014). Infection control for norovirus. Clin. Microbiol. Infect. 20, 731–740. 10.1111/1469-0691.12674 24813073 PMC4624335

[B3] EFSA and ECDC (2021). The European union one health 2019 zoonoses report. EFSA J. 19:e06406. 10.2903/j.efsa.2021.6406 33680134 PMC7913300

[B4] Teunis P. F. M. Moe C. L. Liu P. Miller S. E. Lindesmith L. Baric R. S.. (2008). Norwalk virus: how infectious is it? J. Med. Virol. 80, 1468–1476. 10.1002/jmv.21237 18551613

[B5] WHO (2015). WHO estimates of the global burden of foodborne diseases. Foodborne Diseases Burden Epidemiology Reference Group 2007–2015. Geneva: WHO Press.

